# BAFF and APRIL Receptors in B Cell Immunity and Autoimmunity

**DOI:** 10.1111/imr.70170

**Published:** 2026-09-17

**Authors:** Daisy H. Luff, Edina Schweighoffer, Victor L. J. Tybulewicz

**Affiliations:** ^1^ The Francis Crick Institute London UK

**Keywords:** APRIL, autoimmunity, BAFF, BAFFR, BCMA, common variable immunodeficiency, marginal zone B cells, TACI

## Abstract

BAFF and APRIL are TNF superfamily proteins that bind to BAFFR, TACI and BCMA, members of the TNF receptor superfamily. These proteins have both unique and overlapping roles in B cell development and survival and are major therapeutic targets for antibody‐ and B‐cell‐driven pathologies. BAFF and BAFFR are required for development and survival of follicular and marginal zone (MZ) B cells, whereas BAFF and APRIL acting through TACI and BCMA support plasma cell survival. TACI and BCMA can both be cleaved to generate soluble decoy receptors binding to BAFF or APRIL, forming feedback circuits. Thus, loss of TACI leads to an increase in BAFF, resulting in B cell hyperplasia. Recent work showed that TACI is required for MZ B cell development, a finding that has implications for understanding immune dysfunction in humans. Both monoallelic and biallelic loss‐of‐function TACI mutations result in immunodeficiency, potentially due to impaired MZ B cell function. Paradoxically, monoallelic TACI mutations predispose to autoimmunity. We propose this may be due to increased BAFF levels which promote selection of self‐reactive B cell clones into the mature B cell pool, particularly the MZ B cell compartment. A deeper understanding of this ligand‐receptor system is essential for effective therapeutic targeting.

**Abbreviations**
ADCC: antibody‐dependent cellular cytotoxicity
APRIL: A proliferation inducing ligand
BAFF: B cell Activator of the TNF family
BAFFR: BAFF receptor
BCDT: B cell depletion therapy
BCMA: B cell maturation antigen
BCR: B cell antigen receptor
BiTE: bispecific T cell engaging antibody
CAR: chimeric antigen receptor
CVID: common variable immunodeficiency
FO: follicular
GC: germinal centre
Ig: immunoglobulin
MBC: memory B cell
MM: multiple myeloma
MS: multiple sclerosis
MZ: marginal zone
PB: plasmablast
PC: plasma cell
PI3K: phosphoinositide 3‐kinase
PIP_3_
: phosphatidylinositol (3,4,5)‐trisphosphate
SjD: Sjogren's Disease
SLE: systemic lupus erythematosus
TACI: Transmembrane activator and CAML interactor
TD: T‐dependent
TI: T‐independent
TNF: tumor necrosis factor
TNFRSF: TNF receptor superfamily
TNFSF: TNF superfamily

## 1 Introduction

### 1.1 BAFF and APRIL

**FIGURE 1:**
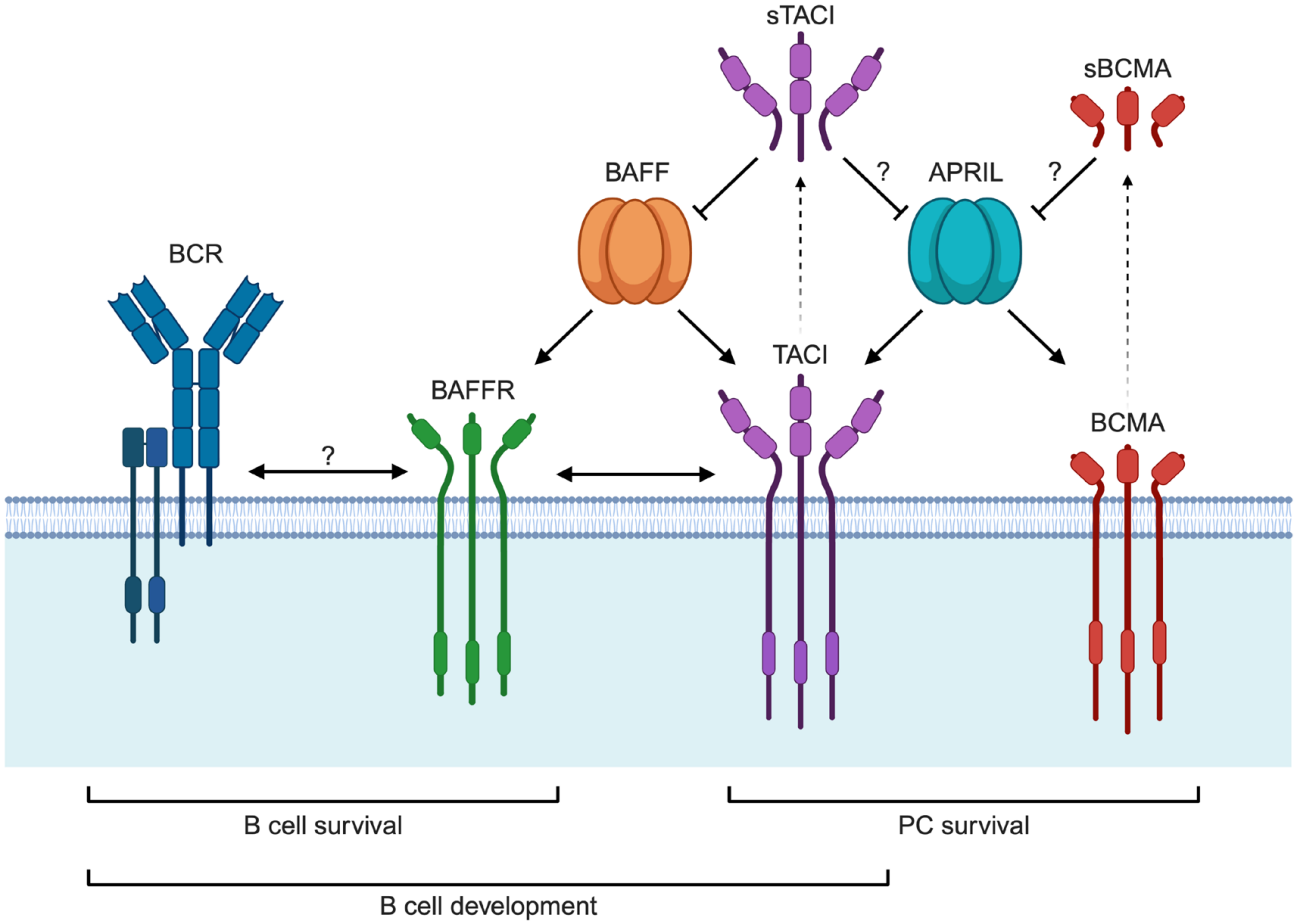
Schematic of the functions and interactions of BAFF and APRIL receptors. BAFF binds BAFFR and TACI, while APRIL binds TACI and BCMA. BAFF binds BCMA with lower affinity than APRIL, and no function has been ascribed to BAFF‐BCMA signaling to date. BAFF‐BAFFR signaling and BCR signaling are both essential for B cell survival and mature B cell development. TACI signaling is required for MZ B cell development. BAFF‐TACI, APRIL‐TACI and APRIL‐BCMA signaling regulates PC survival. TACI and BCMA are cleaved independently of ligand binding to generate soluble TACI (sTACI) and soluble BCMA (sBCMA), which can act as decoy receptors that neutralize BAFF and APRIL. sTACI likely regulates the level of BAFF, as loss of TACI leads to an increase in serum BAFF in vivo. It has not yet been demonstrated whether sBCMA regulates APRIL levels in vivo. TACI and BAFFR interact in a BAFF‐dependent manner. BAFFR survival signaling requires expression of the BCR, though it is not yet known how these receptors interact. Arrows indicate interactions. Dashed arrows indicate cleavage of receptor extracellular domains from the plasma membrane. Flat‐headed lines indicate negative regulation of soluble ligand levels. Question marks indicate unknowns. Figure made using Biorender.

BAFF (B cell Activator of the TNF family, TNFSF13B, CD257) and APRIL (A proliferation inducing ligand, TNFSF13, CD256) are two closely related members of the TNF superfamily that share significant sequence homology, particularly in their TNF homology domain (THD) that mediates trimerization of ligand monomers as well as receptor binding. BAFF and/or APRIL bind to three related receptors, the Type III membrane proteins and members of the TNF receptor superfamily (TNFRSF): BAFF binds to BAFFR (BAFF receptor, TNFRSF13C, CD268) and TACI (Transmembrane activator and CAML interactor, TNFRSF13B, CD267) whereas APRIL binds TACI and BCMA (B cell maturation antigen, TNFRSF17, CD269) [1] (Figure 1). BAFF binds to monomeric BCMA with very low affinity but has a nanomolar affinity to dimeric BCMA, suggesting that BAFF‐BCMA interaction may occur in vivo where either the ligand or receptor are multimerised [1].

BAFF is expressed as a Type II membrane‐bound protein at the plasma membrane that can be cleaved by a furin protease at a multi‐basic sequence motif to generate a soluble trimeric ligand [1]. BAFF is active in both its membrane‐bound and soluble forms, though in vitro experiments suggest that oligomeric BAFF, such as 6‐mers, which may mimic the membrane‐bound form, may be more efficient at activating TACI compared to soluble trimeric BAFF [2, 3, 4]. BAFF‐deficient mice show a profound loss of mature B cells, a phenotype that can be reversed by administration of soluble BAFF trimers [5]. In contrast, transgenic mice expressing mutant BAFF that cannot be cleaved appear indistinguishable from BAFF‐deficient mice, indicating that soluble BAFF is the predominant form that supports B cell survival in vivo, although it is not sufficient to restore normal levels of surface CD23 expression, for which both soluble and membrane‐bound BAFF are required [5]. Structural analyses of BAFF revealed an unusual “flap” region, formed by a long loop between two β‐sheets (D and E) that mediates interaction between BAFF ligand trimers but makes no contact with the receptors [6]. Flap mutations in human (E223K) and mouse (E247K) BAFF abolish BAFFR signaling activity, despite preserving receptor binding, and mice with the knock‐in E247K mutation phenocopy BAFF‐deficient mice in having severely reduced numbers of B cells, illustrating the importance of trimer‐trimer interactions [7]. Taken together, these results imply that B cell survival is maintained by soluble BAFF trimers that interact with each other through flap regions. Human BAFF trimers can also use this flap to assemble into cage‐like 60‐mers that may increase BAFF potency [4]. However, careful analysis of human serum and CSF, including that from patients with very high BAFF levels, found no detectable BAFF 60‐mers [8]. This could be due to the presence of an as yet unidentified dissociating activity in human serum that prevents formation of BAFF 60‐mers [8]. Notably, mouse BAFF cannot form 60‐mers, likely due to the presence of an additional 30 amino acid residues at the N‐terminus compared to human BAFF [7]. Thus, the predominant form of soluble BAFF present systemically in both humans and mice is trimeric and the physiological relevance, if any, of 60‐mer BAFF in humans remains to be determined.

APRIL is synthesized as a Type II transmembrane protein but is efficiently cleaved by a furin convertase in the trans‐Golgi network and is therefore predominantly secreted in its soluble form [9]. APRIL can, however, bind to cell‐surface heparan sulphate proteoglycans (HSPG) via a basic region distinct from its receptor‐binding site, which may allow capture of APRIL to increase the avidity of binding to TACI and BCMA [10, 11]. While BAFF and APRIL predominantly exist as homotrimers, heterotrimers between BAFF and APRIL can and do form in vivo, which can activate TACI similarly to the homotrimers, but may functionally antagonize BAFFR signaling [12, 13].

### 1.2 BAFF and APRIL Receptors

Unusually for TNF family receptors, BAFFR and BCMA each contain only one cysteine‐rich domain (CRD), which mediates ligand binding, whereas TACI possesses two CRDs but only one (CRD2) is necessary and sufficient for ligand binding [14]. Via these single CRDs, each receptor monomer binds to one monomer within the ligand trimers [15]. Recent cryo‐EM analyses of lipid monolayer‐bound receptor‐ligand complexes suggest that binding of trimeric BAFF induces trimerization of receptors followed by the formation of pentagonal clusters of receptor‐ligand complexes [16]. This pentagonal arrangement is mediated by the interaction of flap regions in neighboring BAFF trimers, without contribution from the receptor. Similar clustering is produced following soluble BAFF binding to BAFFR, TACI or BCMA extracellular domains, and upon membrane‐bound BAFF binding to the BAFFR. Interestingly, binding of TRAF3, the only known interactor of BAFFR, which allows activation of the non‐canonical NF‐κB pathway, induces a shift to hexagonal clustering of BAFF:BAFFR complexes. This is hypothesized to maximize TRAF3 binding, though further investigation is needed to confirm the role of this clustering in downstream signaling, and to determine whether APRIL, which lacks the flap region, mediates similar higher order clustering of TACI and BCMA.

All three receptors can be cleaved to produce a soluble fragment comprising their extracellular ligand binding domain. Both TACI and BCMA are cleaved independently of ligand‐binding, by ADAM10 and γ‐secretase, respectively [17, 18]. The resultant soluble receptor fragments (sTACI and sBCMA) can both bind APRIL, while sTACI also binds BAFF, and they may act as soluble decoy receptors, binding to and thus reducing ligand availability (Figure 1). In contrast, BAFFR cleavage by ADAM10 is dependent on BAFF binding; therefore, sBAFFR is generated while already bound to ligand and is unlikely to serve as a decoy receptor [19].

### 1.3 Expression of BAFF and APRIL and Their Receptors

BAFF is expressed in myeloid cells, T cells and stromal cells; however, BAFF produced by stromal cells is both necessary and sufficient to support normal B cell numbers and serum BAFF levels [20, 21, 22, 23, 24]. Fibroblastic reticular cells (FRC) in the lymph nodes and spleen are major sources of local BAFF, as ablation of FRCs led to loss of BAFF and consequently B cells in secondary lymphoid organs without affecting total serum BAFF levels [25].

APRIL is produced by myeloid cells in both human and mouse bone marrow, starting at the promyelocyte stage [26]. Granulocytes, especially neutrophils, are major producers, but monocytes, eosinophils, megakaryocytes, and osteoclasts also produce APRIL [26, 27, 28, 29].

BAFFR is expressed on all B cells from the transitional Type 1 (T1) stage of B cell development onwards, with low expression on T1 B cells and higher expression on T2 B cells and all subsets of mature B cells—follicular (FO) B cells, marginal zone (MZ) B cells and B1 cells [30, 31]. Following activation, BAFFR is still expressed on germinal centre (GC) B cells and both IgG1+ and IgM+ memory B cells (MBCs); however, expression is lost on plasma cells (PCs) [32].

TACI is expressed from the T1 and T2 transitional B cell stages, with low levels on FO B cells, increased levels on MBCs, higher expression on MZ B cells and highest on PCs [32, 33]. In humans, TACI is expressed on MZ B cells, MBCs and PCs, and can be induced upon B cell activation [34].

A recently developed BCMA:Tomato reporter mouse revealed BCMA expression exclusively in antibody‐secreting cells, that is, plasmablasts (PBs) and PCs, that increases with their maturation [35]. The highest level of BCMA transcription and tomato reporter expression was found on IgA+ PCs compared to PCs expressing other Ig isotypes. In humans, low BCMA expression can be seen on tonsillar CD27+ MBCs, and BCMA expression increases as B cells differentiate into PCs [34]. In addition, malignant PCs of multiple myeloma patients show very high BCMA expression [36].

## 2 BAFF and BAFFR in B Cell Survival

Studies in mice have revealed the critical role of BAFF and BAFFR in B cell survival and homeostasis. While BAFF‐deficient mice have normal numbers of T1 B cells, they show a profound loss of immature T2 B cells, mature FO B cells and MZ B cells [37, 38]. This is accompanied by a strong reduction in serum immunoglobulins of each class, except IgA. T‐independent (TI) and T‐dependent (TD) B cell immune responses are also greatly impaired. Interestingly, B1 cells are present in normal numbers [37, 38]. Such a loss of B cells could be due to a requirement for BAFF in development beyond the T1 stage, or a role for BAFF in B cell survival in T2, FO and MZ B cells. The latter possibility is supported by studies in which mice were treated with anti‐BAFF antibodies or with fusion proteins consisting of the extracellular domains of BAFFR or TACI fused to the Fc domains of immunoglobulins (BAFFR‐Ig and TACI‐Ig), resulting in a rapid loss of T2, FO and MZ B cells [38, 39, 40]. In contrast to BAFF‐deficient mice, BAFF‐overexpressing transgenic mice have an expanded mature B2 and B1 cell pool, presumably due to increased survival of these cells, with an increased proportion of MZ B cells [41]. BAFF is also required for the survival of human B cells, as evidenced by the reduction of naive B cells in the blood of systemic lupus erythematosus (SLE) patients treated with belimumab, an anti‐BAFF antibody [42, 43].

BAFFR‐deficient mice show a reduction in B cell numbers similar to that seen in mice lacking BAFF, with a large reduction in T2 B cells, FO B cells and MZ B cells, but no change in the numbers of T1 B cells or B1 cells [44, 45]. Furthermore, treatment of mice with an anti‐BAFFR antibody that blocks BAFF binding to BAFFR, or an inducible genetic deletion of the *Tnfrsf13c* gene encoding BAFFR, results in a similar loss of T2, FO and MZ B cells, but has no effect on B1 cells [31, 32]. The phenotypic similarity of mice deficient in BAFF or BAFFR and the effects of blocking BAFF or BAFFR suggest that for the survival of B cells beyond the T1 stage, BAFFR is the critical receptor for BAFF. In addition to its role in B cell survival, it remains possible that BAFF signaling through BAFFR is also required for B cell development from the T1 B cell stage through the T2 stage and into the mature FO and MZ B cell compartments. In line with the large reduction in mature B cell numbers, BAFF‐ and BAFFR‐deficient mice have strongly decreased TD B cell responses [45]. However, TI Type II (TI‐II) responses to repetitive polysaccharide antigens are much more affected in BAFF‐deficient mice than in the absence of BAFFR, suggesting that one or both of the other two receptors, TACI and BCMA, may contribute to this immune response [45]. In addition, while CD21 levels are equally affected by loss of BAFF or BAFFR, CD23 is more affected in the absence of BAFF, again suggesting that some BAFF functions may be mediated by receptors other than BAFFR [45].

Earlier studies had concluded that MBCs do not express BAFFR and that blocking BAFF binding to BAFFR in mice did not affect MBC numbers, implying that the BAFF‐BAFFR pathway was not required for MBC survival [46]. However, another study showed that blocking BAFF resulted in a decrease in IgM+ but not IgG1+ MBCs [47]. In a more recent study, we showed that both IgM+ and IgG1+ MBCs express *Tnfrsf13c* (BAFFR) RNA and cell surface BAFFR protein [32]. Furthermore, this study along with another showed that blocking BAFF or inducibly deleting BAFFR resulted in loss of both IgM+ and IgG1+ MBCs in spleen, bone marrow and lung, demonstrating a requirement for BAFF and BAFFR in MBC survival, similar to that seen for naive B cells [32, 48]. This difference in results may be due to, in the more recent studies, the use of antigen‐specificity to identify MBCs and the use of longer time points to establish a requirement for BAFF and BAFFR in long‐term maintenance of MBCs.

Human MBCs also express BAFFR, at levels similar to those on naive human B cells, suggesting that human MBCs may also require BAFF and BAFFR for survival [49]. However, while treatment of SLE patients with belimumab (anti‐BAFF) results in a loss of naive B cells, it causes a transient increase in MBCs in the blood, suggesting that BAFF may not be required for human MBC survival [42, 43]. In support of this, in vitro BAFF treatment supported survival of naive human B cells but not MBCs, and induced fewer transcriptional changes and much weaker phosphoinositide 3‐kinase (PI3K) activation in human MBCs compared to naive B cells, possibly due to weaker interactions between BAFFR and the B cell antigen receptor (BCR) on MBCs [49]. In contrast, loss of BAFFR affects the in vitro survival of both human naive and MBCs to a similar extent, suggesting that BAFFR may have a BAFF‐independent function in the survival of human MBC [49]. One caveat to these conclusions is that most MBCs reside in tissues and not in the blood, and numbers of tissue‐resident MBCs have not been evaluated in patients treated with belimumab. It is possible that belimumab impacts on the BAFF‐expressing survival niche in tissues, resulting in release of MBCs into the blood. Further work is needed to examine the roles of BAFF and BAFFR in tissue‐resident human MBCs.

## 3 BAFF‐BAFFR Signaling

Unlike most TNFRSF receptors, the intracellular portion of BAFFR does not have a death domain. In contrast, BAFFR belongs to a subgroup of TNFRSF receptors with TRAF‐interacting motifs. BAFFR has a single PVPAT sequence in the intracellular domain that binds TRAF3 [50]. In the absence of BAFF‐BAFFR binding, TRAF3 is in a complex with TRAF2, cellular inhibitor of apoptosis proteins (cIAP1 and cIAP2), and NF‐κB‐inducing kinase (NIK). This allows continuous cIAP‐mediated K48‐linked ubiquitination and proteasomal degradation of NIK, resulting in barely detectable steady‐state levels of the NIK protein. Following BAFF binding to BAFFR, the receptor recruits the TRAF2‐TRAF3‐cIAP1/2‐NIK complex by binding to TRAF3. This leads to re‐targeting of the cIAP1/2‐mediated K48‐linked ubiquitination to TRAF3 itself, resulting in TRAF3 degradation, release of NIK from the complex, accumulation of NIK and downstream signaling [51, 52, 53]. NIK then activates the non‐canonical NF‐κB pathway by phosphorylating and activating IκB kinase 1 (IKK1), which in turn phosphorylates p100 NFκB2. Once phosphorylated, p100 is partially processed by the proteasome, generating p52 that heterodimerizes with RELB, enters the nucleus and drives transcription of pro‐survival genes such as *Bcl2* and *Mcl1*.

While the non‐canonical NF‐κB pathway is the most well‐studied signaling process activated by BAFF binding to BAFFR, other pathways also play important roles. Notably, inducible deletion in mature B cells of the *Chuk* gene, which encodes IKK1, does not affect their survival, thus BAFFR must signal via alternative pathways to support B cell survival [54]. We and others showed that treatment of B cells with BAFF induces the phosphorylation of CD79A, a signaling subunit of the BCR, and the SYK tyrosine kinase [49, 55]. We found that BAFF‐induced phosphorylation of SYK is dependent on the presence of the BCR, demonstrating cooperation between the BCR and a BAFF receptor, presumably BAFFR, with BAFFR signals routed via the BCR [55] (Figure 1). Recent studies have shown that BAFFR and BCR interact directly, which may be important to facilitate this receptor crosstalk [49]. The BCR‐ and SYK‐mediated signal from BAFFR activates the ERK1 and ERK2 kinases and the PI3K pathway, and in genetic rescue experiments we showed that SYK transduces signals via ERK and PI3K which support B cell survival [55]. The BAFF‐induced BCR‐dependent activation of PI3K may be transduced via CD19, which is phosphorylated on its intracellular tail following addition of BAFF, providing a docking site for PI3K [54]. PI3K signaling leads to AKT and mTORC1 activation which can support metabolic fitness and contribute to survival by maintaining MCL1 expression [55, 56, 57, 58].

Another possible connection between BAFFR and the BCR is via the BTK kinase, which contributes to the activation of the classical NF‐κB pathway downstream of both receptors [59]. Since the *Nfkb2* gene, encoding p100, is a known target of the classical NF‐κB pathway, BAFFR and BCR signaling via BTK is responsible for maintaining p100 levels that can be processed into p52 via the non‐canonical NF‐κB pathway downstream of the BAFFR [59, 60]. Finally, the MEK5 and ERK5 MAP kinase signaling pathway has also been shown to contribute to BAFFR‐dependent B cell survival, independently of the PI3K and NF‐κB pathways [61].

## 4 TACI in B Cell Survival

The first studies of germline TACI‐deficient mice discovered that TACI loss resulted in a large expansion of mature B cell numbers [45, 62, 63]. This led to the conclusion that TACI was a negative regulator of mature B cell survival, although a B cell‐intrinsic signaling mechanism to explain this was not established. However, later studies observed that TACI‐deficient animals have elevated levels of circulating soluble BAFF in their serum [4, 64]. Given that BAFF overexpression leads to an expansion of mature B cells [41], it seemed likely that B cell hyperplasia in TACI‐deficient mice was caused by the increased BAFF and resultant BAFFR survival signaling, and, importantly, we hypothesized that this confounding factor may mask a cell‐intrinsic phenotype of TACI deficiency.

To circumvent the issue of increased BAFF in germline TACI‐deficient mice, we used mixed bone marrow chimeras to study the cell‐intrinsic function of TACI by reconstituting the immune system of irradiated RAG1‐deficient mice with TACI‐deficient (KO) or wild‐type (WT) bone marrow mixed 1:1 with allotypically‐marked WT marrow [33]. Comparing TACI‐deficient B cells in KO:WT chimeras with WT B cells in WT:WT chimeras, which had equivalent levels of circulating BAFF, we found that TACI‐deficient B cells were not expanded in number. Furthermore, using continuous EdU labelling in vivo we demonstrated that TACI loss did not affect the turnover, and therefore the survival, of transitional or mature B cells. Together, these observations confirmed that TACI does not regulate B cell survival in a cell‐intrinsic manner. It is important to note, however, that TACI likely plays a cell‐extrinsic role in regulating systemic and local BAFF levels and therefore, indirectly, B cell survival. Mechanistically, this cell‐extrinsic role of TACI may be due to ADAM10‐induced cleavage of TACI which releases sTACI, a decoy receptor that binds and neutralizes BAFF [17]. The inverse correlation between B cell numbers and serum BAFF concentration in mice and humans may therefore reflect the combined effect of BAFFR binding and internalizing BAFF and sTACI neutralizing BAFF [65].

## 5 Mature B Cell Development and the Role of BAFFR

B cell development is initiated in the bone marrow where pro‐B and then pre‐B cells undergo immunoglobulin heavy chain and then light chain rearrangement, respectively, generating immature B cells with a BCR consisting of surface‐bound IgM [66, 67]. These immature B cells leave the bone marrow and migrate through the blood to complete their maturation in the spleen as transitional T1 and T2 B cells. Both immature bone marrow B cells and transitional B cells have a short lifespan of 2–3 days and the majority do not become mature B cells [68, 69]. The fate of transitional B cells is dependent on the strength of BCR signaling. Those receiving too little BCR signal die by neglect, whereas too strong BCR signaling, indicative of autoreactivity, leads to negative selection and elimination by apoptosis. Entry of T2 B cells into the mature FO and MZ B cell compartments requires positive selection through recognition of endogenous antigen by the BCR, with the choice between FO and MZ fates depending, at least in part, on the strength of BCR signaling received [70]. Studies using the Thy1‐specific (ATA) transgenic BCR model and a Nur77‐eGFP reporter of BCR signaling strength revealed that the FO fate is acquired by cells with a broad range of self‐reactivity whereas the MZ compartment has a relatively higher positive selection threshold and lower negative selection threshold, resulting in a higher and narrower range of self‐reactivity in MZ B cells compared to FO B cells [71, 72].

In line with the role of BCR signal strength in the regulation of mature B cell development and the MZ versus FO fate, the BCR co‐receptor CD19, which is a positive regulator of BCR signaling, also plays a critical role at this stage. Mice lacking CD19 have reduced numbers of mature B cells, but specifically lack the MZ B cell population, indicating that the lower levels of BCR signaling in the absence of CD19 are insufficient for positive selection into the MZ B cell population [73, 74, 75]. Mechanistically, activation of PI3K signaling by the BCR‐CD19 complex is the key pathway required for MZ B cell development, through CD19‐mediated recruitment of PI3Kδ, the predominant class IA PI3K isoform in B cells, which is also required for MZ B cell development [76, 77, 78, 79].

In addition to the BCR and CD19, BAFFR is required for the maturation of transitional B cells. Constitutive BAFFR deficiency results in a near‐complete block in B cell development at the T1 stage, indicating that BAFFR‐mediated survival signals are required for T2 B cells to survive long enough for positive selection into the mature B cell compartments [44]. In line with this, transgenic overexpression of the anti‐apoptotic protein BCL2 in B cells restores the immature T2 and mature FO B cell compartments in BAFFR‐deficient mice [44]. However, BCL2 overexpression was not sufficient to restore MZ B cells, suggesting that BAFFR may also play a survival‐independent role in MZ B cell development [44, 80].

## 6 TACI Is Required for MZ B Cell Development

**FIGURE 2:**
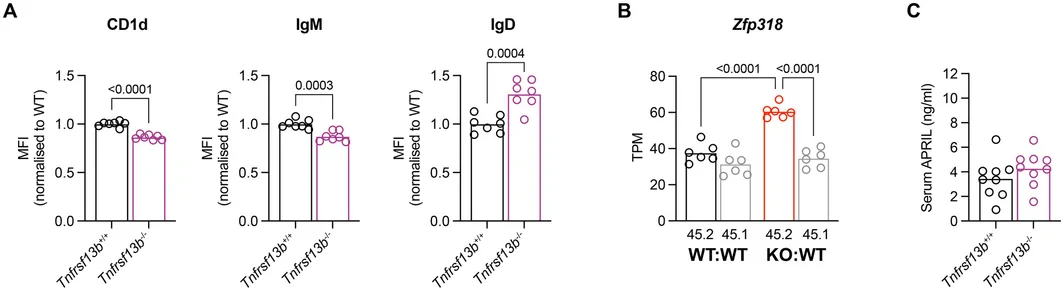
TACI is required for MZ B cell development. (A) CD1d, IgM and IgD surface expression on MZ B cells (CD1d^hi^IgM^hi^CD93^‐^B220^+^CD19^+^) from *Tnfrsf13b* ^+/+^ (WT) and *Tnfrsf13b* ^−/−^ (TACI‐deficient) mice, quantified as the geometric mean fluorescence intensity (MFI) and normalized to the mean MFI of WT within the same experiment. (B) *Zfp318* mRNA expression in MZ B cells from TACI KO:WT and TACI WT:WT chimeras, determined by RNAseq and quantified as transcripts per million (TPM) [33]. Black: WT CD45.2^+^; red: TACI KO CD45.2^+^; gray: WT CD45.1^+^. (C) Concentration of APRIL in the serum of *Tnfrsf13b* ^+/+^ (WT) and *Tnfrsf13b* ^−/−^ (TACI‐deficient) mice, measured by ELISA as described in [33] except using kit ABIN6970939. Bars show the mean of data pooled from 2 independent analyses. Each point represents one mouse. Statistical tests: Unpaired t test (A, C), repeated measures two‐way ANOVA with Fisher's LSD test (B). Numbers above graphs indicate *p*‐values where *p* ≤ 0.05, otherwise no value is shown. All methods as previously described [33].

Our recent work identified TACI as an additional critical regulator of MZ B cell development [33]. Using mixed bone marrow chimeras to study the cell‐intrinsic effect of TACI deficiency, without the confounding influence of elevated BAFF levels in germline TACI‐deficient animals, revealed that loss of TACI causes a selective reduction in the number of MZ B cells, but not the FO or transitional B cell populations. The residual immature MZ B cell population formed in the absence of TACI is phenotypically and transcriptionally less MZ‐like and more FO‐like, demonstrating that TACI regulates the MZ versus FO fate decision and is required for the maturation of MZ B cells (Figure 1). We also found that TACI‐deficient B cells are unable to mount an IgM antibody response to the TI‐II antigen, TNP‐Ficoll [33]. This may be due to the defective development of the MZ B cell population, which, along with B1 cells, are the primary responders to T‐independent antigens [81, 82]. Interestingly, germline TACI‐deficient mice also generate impaired antibody responses to TNP‐Ficoll, despite having expanded mature B cell numbers [62, 63]. When we analyzed their B cell compartment in more detail, we found that TACI‐deficient mice have a reduced proportion of MZ B cells, indicative of a selective MZ defect [33]. Furthermore, MZ B cells in these germline TACI‐deficient mice have reduced surface levels of IgM and CD1d and increased levels of IgD, making them less MZ‐like and more FO‐like, a phenotype we also observed in TACI‐deficient MZ B cells in the mixed chimeras (Figure 2A) [33]. Thus, TACI is required for MZ B cell development, even in the presence of elevated BAFF, and this may explain the impaired TI‐II antibody responses in germline TACI‐deficient mice.

Interestingly, transcriptomic analysis revealed that *Zfp318* expression is increased in TACI‐deficient MZ B cells in the mixed chimeras (Figure 2B). ZFP318 is a critical positive regulator of IgD expression promoting transcription of the *Ighm‐Ighd* heterogenous RNA that is alternatively spliced to generate *Ighd* mRNA [83, 84]. This result suggests that TACI supresses *Zfp318* expression during the maturation of MZ B cells to promote the IgD^lo^ IgM^hi^ phenotype characteristic of this subset. In line with this, *Zfp318* expression is higher in FO B cells than in MZ B cells [84].

We found that TACI regulates MZ B cell development at least in part through activation of the PI3Kδ‐AKT pathway, leading to inhibition of FOXO1 and decreased expression of *Klf2*, two transcription factors that are known to inhibit MZ B cell development [33, 85, 86, 87]. Since the BCR and CD19 also recruit and activate PI3Kδ [88, 89, 90], our results suggest that TACI, the BCR and CD19 all converge on PI3Kδ, which generates the second messenger phosphatidylinositol (3,4,5)‐trisphosphate (PIP_3_), to promote MZ B cell development.

We found that TACI associated with PI3Kδ in mature B2 cells in a BAFF‐ and APRIL‐stimulation‐independent manner [33]. This does not preclude the possibility that the interaction of TACI with PI3Kδ is further induced upon BAFF or APRIL stimulation, perhaps when transitional B cells receive simultaneous BCR signals. Indeed, given that PI3Kδ requires binding to phosphorylated tyrosine residues for its activation, and TACI does not contain such motifs, our observation raises the possibility that TACI may co‐operate with the BCR signaling pathway. Specifically, TACI may act as a scaffold for adaptor proteins that are phosphorylated by BCR‐induced kinases and recruit PI3Kδ in a similar fashion to the role of CD19, which is directly phosphorylated by Lyn following BCR engagement to enable PI3Kδ recruitment and activation at the plasma membrane [91, 92]. Further investigation is required to delineate the molecular mechanisms by which TACI associates with PI3Kδ.

The key, if not sole, PIP_3_‐regulated PI3Kδ effector required for MZ B cell development is the kinase AKT. This is based on the observations that deletion of *Akt1* and *Akt2* or mutation of the inhibitory AKT phosphorylation sites on FOXO1 both result in a near complete loss of the MZ B cell population, while constitutive activation of AKT1 in B cells is sufficient to produce a mature B2 compartment that is ~90% MZ‐like [86, 93]. Another receptor that selectively regulates the maturation of MZ B cells is NOTCH2 [94, 95]. Interestingly, there is some evidence that NOTCH signaling may indirectly regulate the PI3K‐AKT pathway; however, this has not been demonstrated in B cells [96, 97, 98]. Further work is required to investigate potential crosstalk between NOTCH2 and PI3Kδ‐AKT signaling in B cells.

Another outstanding question concerns the relative importance and non‐redundant versus redundant roles of TACI, NOTCH2, BCR and CD19 in MZ B cell development. In transcriptomic analyses of TACI‐deficient MZ B cells, we did not find dysregulation of NOTCH transcription factor activity, indicating that TACI is not required for NOTCH2 activity [33]. Previous studies demonstrated that overexpression of the NOTCH2 intracellular domain (ICD), which acts as a constitutively active transcription factor, in CD19‐deficient B cells leads to a decrease in *Foxo1* and *Klf2* expression, suggesting that NOTCH2 and TACI pathways converge on the same transcription factors to regulate MZ B cell development [99]. Furthermore, the observation that NOTCH ICD overexpression can generate MZ B cells in the absence of CD19 suggests that NOTCH2, the BCR and CD19 also converge on the same pathways [95]. In line with this, the loss of MZ B cells is more severe in *Notch2* ^fl/−^ CD19‐Cre (CD19^+/−^) mice than in *Notch2* ^fl/−^ Mx1‐Cre mice (CD19^+/+^), indicating that CD19 can provide signals for partial but not complete MZ B cell development in the absence of NOTCH2 [94]. This is similar to the residual, immature MZ population seen in the absence of TACI in our mixed chimera model, where CD19 and NOTCH2 could provide signals for partial MZ development [33]. It is possible that all four receptor pathways synergise to provide sufficient FOXO1 inhibition for MZ B cell development. It remains to be seen what non‐redundant signals BCR, CD19, TACI and NOTCH2 may provide for generation of the MZ compartment.

MZ B cells express higher levels of TACI compared to FO B cells, leading to the question of how TACI expression is regulated to increase its expression in transitional B cells destined to become MZ B cells. TACI surface expression can be induced by IgM crosslinking in transitional B cells in vitro [100], and correlates with the level of BCR signaling in transitional B cells in vivo, as indicated by Nur77‐GFP reporter expression, which is proposed to reflect engagement of the BCR by endogenous antigens [70, 101]. Thus, transitional B cells receiving higher levels of BCR signaling, due to higher affinity for endogenous antigen, may induce expression of TACI which then promotes entry into the MZ compartment. However, the FO and MZ compartments appear to tolerate overlapping levels of endogenous BCR signaling and self‐reactivity [70], indicating that qualitative as well as quantitative differences in BCR signaling determine TACI‐driven MZ entry. Indeed, the BCR repertoire in MZ B cells is thought to be enriched for polyreactive antigen receptors that recognize conserved microbial patterns. However, an unbiased analysis of the antigen‐specificity of the BCR repertoire in MZ B cells is lacking and the endogenous antigens that may promote MZ B cell development remain unknown [102]. Notably, gut microbiota are not essential for MZ B cell development as MZ B cells are present in germ‐free mice [103, 104]. Co‐stimulatory signals could also have an influence. Stimulation of TLR7, TLR9, or TLR4 can induce TACI expression [105]. However, neither TLR7 nor the TLR adaptor MYD88 are required for TACI expression on transitional B cells, and MYD88 is not required for the development of MZ B cells [100, 104].

TACI is engaged by both BAFF and APRIL, thus either cytokine could be required for TACI‐dependent MZ B cell development. Both APRIL‐deficient mice and transgenic mice overexpressing human APRIL have no defects in B cell development and no change in the numbers of MZ B cells [106, 107]. In contrast, transgenic mice overexpressing BAFF have greatly expanded B cell numbers, with a disproportionately increased MZ compartment in the spleen and the unusual appearance of MZ‐like cells in the blood, peripheral lymph nodes and submaxillary glands [41, 105, 108, 109]. From these observations, we can deduce that BAFF is sufficient for MZ B cell development and may be the predominant ligand inducing TACI signaling during MZ B cell maturation in the spleen. Future work to test this hypothesis will require mutation of BAFF to prevent binding to TACI but not BAFFR.

## 7 Human MZ B Cell Development

The role of TACI in MZ B development is likely to be conserved in humans given the phenotypic, functional and developmental similarities between human and mouse MZ B cells. Human MZ B cells, frequently named “IgM+IgD+ unswitched memory” B cells due to their expression of CD27, also express high levels of TACI, CD21 and CD1c, and higher IgM and lower IgD compared to the naive FO B cell equivalent, as in mice [64, 110]. While similarly localized in the human splenic marginal zone, human MZ B cells can also circulate in the blood and exhibit a low level of somatic hypermutation. This can occur independently of cognate T‐cell help outside of the GC, but can also take place in gut GCs, and is observed in MZ B cells in utero, and therefore likely occurs independently of infection [110, 111, 112, 113].

The development of human MZ B cells from immature precursors can be induced by NOTCH2 signaling in vitro and is dependent on AKT activity, as in mice [86, 114]. The requirement for NOTCH2 activity is supported by the observation that NOTCH2‐haploinsufficient Alagille syndrome patients exhibit a reduction in circulating MZ B cells but not in switched MBCs [114]. Interestingly, transcriptomic analyses of the MZ B cell developmental trajectory in humans demonstrate that *TNFRSF13B* (TACI) expression is initiated in the MZ B cell precursor population, similarly to the timing in mouse transitional B cells [33, 114, 115].

In support of a conserved role for TACI in human MZ B cell maturation, monoallelic and biallelic loss‐of‐function mutations in TACI are associated with the development of common variable immunodeficiency (CVID), a primary antibody deficiency characterized by reduced humoral responses to encapsulated bacteria and polysaccharide vaccines [116, 117]. Thus, the phenotype of TACI deficiency in humans is consistent with an impairment in the MZ B cell population, which provides protection against encapsulated bacterial infections and mounts antibody responses to polysaccharide vaccines [110, 118, 119].

## 8 BAFFR‐TACI Interaction

Using proteomics to identify proteins interacting with BAFFR, we found that TACI and BAFFR interact in a BAFF‐dependent manner in primary mouse B cells [33] (Figure 1). It is not yet clear how this association between the receptors is mediated. However, BAFF trimers can form higher‐order oligomers due to the presence of a loop region, or flap, in each BAFF monomer that can interact with a flap in neighboring trimers, and this may mediate the interaction of ligand‐bound BAFFR and TACI. This could be tested by examining whether BAFF carrying the E247K flap mutation is unable to induce the TACI and BAFFR interaction. Alternatively, TACI and BAFFR may interact directly, either through trimer‐trimer interactions or potentially by forming heterotrimers, albeit in both cases the association would be dependent on BAFF binding to one or both receptors.

A further question concerns the functional consequences, if any, of the BAFFR‐TACI interaction. Our work showed that BAFF‐induced survival signaling is intact in TACI‐deficient B cells in vitro and that TACI‐deficient B cells do not have a survival defect in vivo [33]. Thus, TACI is not required for BAFFR survival signaling. However, it remains possible that BAFFR may support TACI signaling required for MZ B cell development through their interaction. Indeed, the observation that ectopic expression of BCL2 in BAFFR‐deficient mice restored FO but not MZ B cells suggested that BAFFR may play a survival‐independent role in MZ B cell development [44, 80]. Interestingly, we have previously shown that BAFFR co‐opts components of the BCR signaling pathway for survival signaling, including the kinase SYK [55]. It is possible that BAFFR interaction with TACI provides local kinase activity that promotes the association of TACI with PI3Kδ. Thus, although the mechanism by which BAFFR connects to SYK remains unknown, BAFFR, TACI and BCR components may co‐localize at the plasma membrane to activate common downstream pathways, including PI3K‐AKT, during transitional B cell maturation. Future work to investigate the hypothesis that BAFFR supports TACI‐mediated MZ B cell development would require inhibition of the BAFFR‐TACI interaction without interfering with ligand binding to BAFFR or TACI, or affecting BAFFR survival signaling.

## 9 TACI and BCMA in PC Survival

Antibody‐secreting cells are the terminal differentiation stage of the B cell lineage and are specialized to secrete immunoglobulins. While many exist as short‐lived proliferating PBs or non‐proliferating PCs, some migrate to niches in the bone marrow and gut‐associated lymphoid tissues to persist for months or years as long‐lived PCs [120]. TACI is highly expressed at the surface of splenic and bone marrow PBs and PCs, along with BCMA, whereas BAFFR expression is low to negligible on these populations [32, 33, 35]. APRIL and BAFF have been suggested to support the survival of PCs in long‐lived niches [121], but given the overlapping specificity of BCMA and TACI for APRIL, as well as the essential role of BAFF in the development and maintenance of naive B cell precursors, the contribution of each receptor and ligand specifically to PC survival in vivo remained unclear until recently.

Blocking BAFF and APRIL function with antibodies in vivo indicated that there is considerable redundancy between these two cytokines, and either BAFF or APRIL can support the maintenance of a majority (~80%–90%) of the bone marrow, spleen and gut PC populations [122]. The exception to this was bone marrow IgG+ or IgM+ PBs, which did not require BAFF or APRIL, and splenic IgG+ PBs and PCs, which relied on IL6 more than BAFF or APRIL [122]. The same study also combined the ligand‐blocking approach with germline TACI‐deficient and BCMA‐deficient mouse models to determine which ligand‐receptor pairs are important for PC maintenance, revealing that bone marrow PCs and gut IgA PCs rely on BAFF:TACI, APRIL:TACI and APRIL:BCMA signaling [122]. Notably, no requirement for BAFF:BCMA signaling in PC survival was observed and to date no function has been ascribed to this ligand‐receptor pair. Furthermore, the study demonstrated that TACI and BCMA play redundant roles in the maintenance of bone marrow PCs and partially redundant roles in intestinal IgA PCs—that is, in the absence of one receptor, the other is able to compensate to support these PC populations, and their individual contributions are not revealed in single receptor‐deficient mice until BAFF and/or APRIL are also blocked. This is in line with a study of two different BCMA‐deficient mouse models, which found that BCMA was dispensable for PC maintenance in both unimmunised mice and following TD immunization [123]. Nonetheless, despite normal numbers of bone marrow and gut PCs, germline TACI‐deficient mice have reduced serum IgM and IgA, indicating a non‐redundant role for TACI in the generation of these antibody responses [122].

In contrast to the results from TACI‐deficient mice, our study of the cell‐intrinsic role of TACI using mixed chimeras demonstrated that TACI loss leads to a ~50% reduction in splenic PCs, indicating a non‐redundant role for TACI in the generation and/or maintenance of this population in these mixed chimeras [33]. This may result from the impaired maturation and reduced numbers of TACI‐deficient MZ B cells, defects in PC differentiation or PC survival, or a combination of these. In vitro, TACI‐deficient MZ B cells showed only a small defect in PB differentiation, implying that the reduction in splenic PCs is more likely due to impaired TACI‐dependent MZ B cell development or PC survival.

The apparent redundancy between TACI and BCMA in germline knockout mice may be confounded, at least in part, by increased BAFF and/or APRIL levels arising due to the loss of these receptors. Elevated BAFF in germline TACI‐deficient mice expands the number of FO B cells and increases T cell help received by cognate B cells due to elevated ICOSL expression, which together may increase PB and PC generation following TD antibody responses [33, 63, 121, 124]. It is also possible that the loss of sTACI decoy protein may lead to elevated APRIL levels in TACI‐deficient mice that then increase compensatory BCMA signaling. To test this hypothesis, we analyzed the concentration of circulating APRIL in the serum of TACI‐deficient compared to WT mice, but did not observe a significant increase (Figure 2C). However, this does not exclude the possibility that local levels of APRIL in PC survival niches may be increased in the absence of TACI.

Like TACI, the BCMA extracellular domain is also constitutively cleaved from the surface of PCs, though by γ‐secretase, not ADAM10, and the resulting sBCMA protein can act as a decoy receptor that binds to APRIL, regulating the level of this cytokine [18]. Thus, BCMA‐deficient mice may have elevated levels of circulating or local APRIL leading to increased TACI signaling in B cells and PCs. The consequences of this could be skewed mature B cell development towards the MZ fate as well as increased PC survival. Against the former possibility, transgenic mice overexpressing human APRIL in T cells (APRIL‐Tg) have normal mature FO, MZ and B1 B cell development [106]. PC populations have not yet been examined in detail in APRIL‐Tg mice; however, they have increases in serum IgM, IgM antibody responses, and switched‐antibody responses to TI‐antigens [106, 125]. BCMA‐deficient animals exhibit some phenotypes in line with elevated APRIL, including increased serum IgA, increased total PBs and PCs in the mesenteric lymph nodes, and increased bystander IgM and IgA antibody‐secreting cells in the bone marrow and mesenteric lymph nodes following recall responses [123]. Therefore, further studies would be needed to examine the cell‐intrinsic roles of BCMA without the confounding effect of elevated APRIL levels.

Given that TACI is required for MZ B cell development, and BAFF is critical for BAFFR‐mediated naive B cell survival, dissecting the roles of TACI and BCMA in PC survival will require genetic manipulation at the PC stage using conditional knockout alleles and genetic time‐stamping techniques to study PC longevity. Importantly, to avoid the confounding effects of increased BAFF or APRIL concentrations, studies of BAFF and APRIL receptor cell‐intrinsic functions should employ the mixed bone marrow chimera approach or targeted receptor loss‐of‐function mutations that do not inhibit ligand binding.

## 10 BAFF, BAFFR, and TACI in Autoimmunity

It is well established that increased BAFF levels are associated with antibody‐mediated autoimmunity, and consequently there are several existing and emerging therapeutic strategies that target BAFF and BAFFR for the treatment of B‐cell‐driven chronic autoimmune disease, with variable efficacy [126]. Circulating BAFF levels are elevated in a subset of patients with SLE, Sjogren's Disease (SjD) and rheumatoid arthritis [108, 127]. This phenotype is not solely correlative or a consequence of chronic inflammation in these diseases. Indeed, a mutation in the 3′UTR of the gene encoding BAFF that causes increased expression of BAFF protein is associated with a moderately increased risk of developing SLE and multiple sclerosis (MS) [128]. Strong evidence for a causative role of elevated BAFF in the development of autoimmune disease comes from studies of transgenic mouse models overexpressing BAFF. These BAFF‐Tg mice develop a spontaneous, T‐independent lupus‐like disease and nephritis, as well as SjD‐like pathology, with increased anti‐nuclear (ANA) and anti‐ribonucleoprotein (RNP) autoantibodies [41, 105, 108].

The mechanism by which excess BAFF promotes autoimmunity was uncovered using crosses between transgenic mice expressing a hen egg lysozyme (HEL)‐specific BCR (SWHEL), mice expressing soluble HEL (sHEL) and the BAFF‐Tg strain. In the presence of sHEL and normal physiological BAFF levels, autoreactive HEL‐specific B cells become anergic and are eliminated by apoptosis before entering the mature compartments. However, overexpression of BAFF rescues intermediate‐affinity self‐reactive transitional B cells from negative selection at the peripheral tolerance checkpoint, even when these B cells are competing with B cells expressing a polyclonal repertoire of BCRs [109]. This is likely due to the provision of increased pro‐survival signals via BAFFR at the T1 and T2 stages, when BAFFR expression begins. Indeed, autoreactive transitional B cells have a higher dependency on BAFF signals for survival, due to increased expression of the pro‐apoptotic protein BIM and hyporesponsive BCR signaling [129]. In addition to rescuing autoreactive clones from negative selection and thereby predisposing to autoimmunity, excess BAFF is also able to rescue clones that receive insufficient positive selection BCR signals for entry into the mature compartment [130]. Thus, elevated BAFF in effect broadens and “un‐restricts” the mature BCR repertoire. Supporting this view, studies of transgenic mice expressing DNA‐specific BCRs found that BAFF overexpression broadens and diversifies the BCR repertoire, allowing maturation of both high‐ and low‐affinity autoreactive cells [131]. Intriguingly, BAFF overexpression allows self‐reactive immature clones to disproportionately enter the mature MZ population, a compartment from which they are normally excluded [109]. As discussed earlier, entry into the MZ compartment is normally tightly controlled by strict positive and negative selection thresholds [70, 72]. In line with this model of relaxed peripheral tolerance, BAFF‐Tg mice have expanded numbers of FO, MZ, B1a and B1b cells, with a disproportionate increase in MZ B cells in the spleen and the unusual appearance of MZ‐like cells in the blood, peripheral lymph nodes and submaxillary glands [41, 105, 108, 109]. MZ B cells isolated from BAFF‐Tg mice generate anti‐dsDNA antibodies following stimulation through TLR4, TLR7 or TLR9, confirming that BAFF overexpression causes self‐reactive B cell clones to appear in the MZ subset [105].

In light of the role of TACI in regulating mature MZ B cell development, BAFF overexpression may not only provide survival signals via BAFFR, but also developmental signals via TACI at the T1 or T2 stages, when TACI expression is induced. The resulting excess BAFF:TACI signaling may promote maturation of autoreactive transitional B cell clones into the MZ compartment, rather than the FO compartment, which may account for both the disproportionate expansion and the inclusion of self‐reactive clones in the MZ population in BAFF‐Tg mice. We have shown that TACI promotes MZ B cell development through regulation of the PI3Kδ‐AKT‐FOXO1 pathway [33], and expression of hyperactive PI3Kδ leads to a selective expansion of the MZ B cell compartment [78]. Thus, it is notable that in the SWHEL/sHEL mouse model, hyperactive PI3Kδ signaling allows self‐reactive B cells to enter the otherwise forbidden MZ compartment, similarly to the effect of BAFF overexpression [109, 132]. Thus, excess BAFF:TACI signaling caused by elevated BAFF may promote maturation of self‐reactive B cell clones into the MZ fate through activation of the PI3K pathway.

While BAFF regulates the entry of self‐reactive clones into the mature B cell repertoire, overexpression of BAFF can also overcome anergy in high‐affinity self‐reactive B cells, for example in the DNA‐specific 3H9 BCR transgenic model [109, 131]. Increased TACI signaling could also play a role here to enable high‐affinity anergic self‐reactive clones to overcome hyporesponsive BCR signaling, respond to self‐antigen and generate autoantibody responses, perhaps through activation of PI3Kδ signaling. In support of this, PIP_3_, generated by PI3K, has been postulated to regulate the tipping point between peripheral tolerance and autoimmunity [70, 133]. Loss of PTEN or SHIP‐1, negative regulators of PIP_3_ levels, overcomes anergy in mature B cells, indicating that regulation of PI3K signaling is key for maintaining peripheral tolerance [134]. Indeed, PI3Kδ‐hyperactive self‐reactive B cells mount robust responses to self‐antigen stimulation [132]. Therefore, increased signaling through TACI may also contribute to the development of autoimmunity by overcoming anergy in self‐reactive B cells.

Strikingly, loss of TACI protects from the development of lupus‐like autoimmunity and nephritis in BAFF‐Tg mice, with reduced levels of ANA and a loss of switched and unswitched anti‐Sm/RNP antibodies [101, 135, 136, 137]. Loss of TACI does not restore normal FO B cell numbers or reduce the numbers or proportion of MZ B cells in BAFF‐Tg animals, indicating that increased BAFFR signaling is responsible for the B cell hyperplasia in BAFF‐Tg mice and, importantly, that increased survival and consequent expansion of the B cell compartments is not sufficient to promote autoimmunity [101, 136, 137]. These observations suggest that TACI regulates another aspect crucial for the break in peripheral tolerance in BAFF‐Tg animals. Given the role of TACI in MZ B cell development, this could be by promoting the maturation of rescued, autoreactive transitional B cell clones into the “forbidden” MZ compartment, rather than the FO compartment. Indeed, the hyperresponsive T‐independent nature of MZ B cell activation, coupled with high expression of the RNA‐receptor TLR7 and complement receptor CD21, makes the MZ a dangerous population in which to harbor self‐reactive clones [138]. In contrast, the FO compartment may be more able to tolerate self‐reactive clones due to effective peripheral tolerance mechanisms, including downregulation of IgM, which is proposed to possess higher sensitivity to endogenous antigen than IgD, and “clonal redemption” via somatic hypermutation in the germinal centre [70, 72, 139].

TACI‐deficiency in BAFF‐Tg mice normalized the elevated numbers of bone marrow PCs, as well as the greatly increased numbers of splenic PBs and PCs in aged BAFF‐Tg animals [135, 136]. However, since TACI is not essential for PC survival [33, 122], abrogated PC survival may not fully explain the protective effect of TACI loss in BAFF‐Tg mice. Taken together, these observations suggest that TACI may promote the development of autoreactive B cell precursors that then generate autoantibody‐secreting PBs and PCs in BAFF‐Tg mice.

Combined, these observations suggest that therapeutic strategies targeting TACI and BAFF may be able to prevent the entry of self‐reactive clones into the mature B cell compartments, particularly the MZ B cell population. This approach could be used to maintain physiological BAFF levels and TACI signaling to prevent the development of BAFF‐driven chronic autoimmune disease or relapse following B cell depletion therapy. Particular subsets of patients may benefit more than others from this approach; for example, SLE patients with elevated serum anti‐dsDNA IgA2 antibodies, who are more likely to achieve a major clinical response to anti‐BAFF therapy following rituximab‐mediated B cell depletion [140].

## 11 TACI in Immunodeficiency and Associated Autoimmunity

### 11.1 Loss‐of‐function TACI Mutations Lead to Immunodeficiency

The first studies of TACI‐deficient mice demonstrated that TACI is important for the generation of antibody responses to T‐independent, repetitive polysaccharide antigens. TACI‐deficient mice mount reduced IgM, IgG, and IgA responses to TNP‐Ficoll, NP‐Ficoll and pneumococcal polysaccharides (Pneumovax) and exhibit reduced steady‐state antibody levels [62, 63, 122, 124]. Extending this, our studies of TACI‐deficient mixed chimeras revealed that TACI‐deficient B cells have a cell‐intrinsic defect in their ability to generate an IgM response to TNP‐Ficoll [33]. We propose that this defect is at least in part due to the requirement for TACI in the development of mature MZ B cells. Indeed, the MZ subset is key for antibody responses to TI‐II antigens in mice– adoptive transfer studies have demonstrated that MZ B cells, along with B1 cells, but not FO B cells, are the primary responders to T‐I antigens and blood‐borne bacterial antigens [81, 82]. In support of a MZ‐specific role for TACI, early studies of TACI‐deficient mice found TACI to be dispensable for TD antibody responses [62, 63], and our work using TACI‐deficient mixed chimeras showed that TACI‐deficient B cells can generate normal numbers of GC B cells in response to the protein antigen NP‐CGG, in line with unaffected numbers of FO B cells in the absence of TACI [33]. However, other studies of germline TACI‐deficient mice observed a partial reduction in TD antigen‐specific antibodies (IgM, IgG1, IgG2b, and IgG3), which could reflect a defect in the ability of TACI‐deficient GC B cells to upregulate *Blimp1* expression, or a defect in the maintenance of TACI‐deficient antibody‐secreting cells [121, 124]. In line with the former, *Blimp1* expression is repressed by the FOXO1 transcription factor, which our work suggests is inactivated downstream of TACI [33]. Nevertheless, it is clear that TACI is critical both for successful antibody responses to TI antigens, including blood‐borne repetitive polysaccharides and encapsulated bacteria, as well as the development of MZ B cells, a subset specialized in phenotype and localisation to mount rapid responses to such antigens.

**FIGURE 3:**
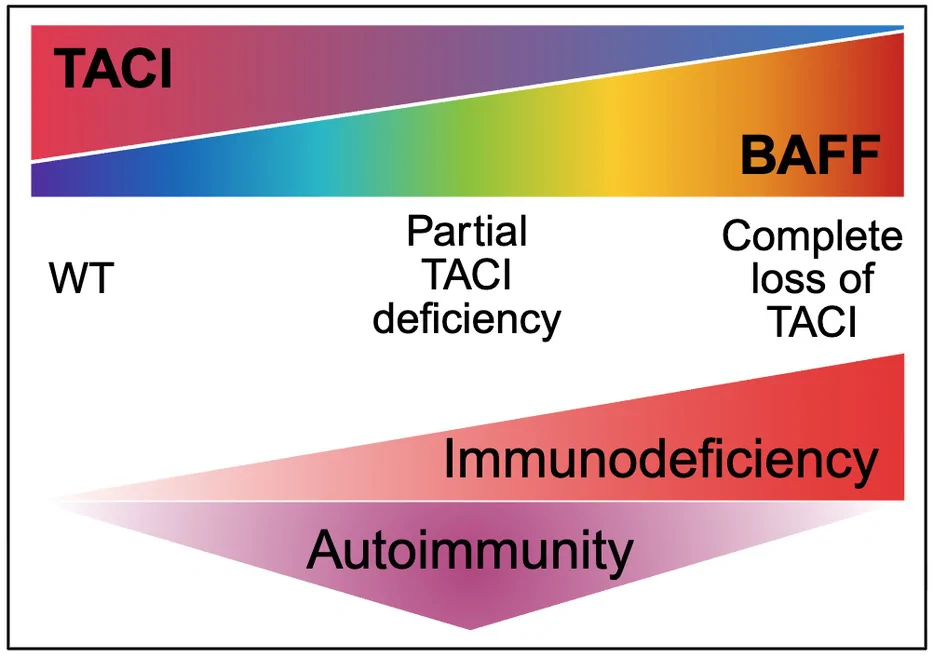
TACI and BAFF balance immunity and autoimmunity. A model for how the levels of functional TACI and BAFF proteins, which are inversely correlated, lead to both immunodeficiency, at least in part through defective MZ B cell development, and autoimmunity, through a breach of peripheral tolerance driven by increased BAFF. Partial TACI deficiency results in the co‐occurrence of autoimmunity and immunodeficiency, whereas complete TACI loss is protective for the development of autoimmunity in the presence of elevated BAFF. Figure made in Biorender.

In a striking human parallel, missense mutations in TACI are a known risk factor for the development of common variable immunodeficiency (CVID). CVID is the most frequent symptomatic primary antibody deficiency, with a prevalence of 1 in 25,000, and is characterized by hypogammaglobulinemia with reduced IgG and often IgA and/or IgM [116]. While CVID presents with broad clinical manifestations, it is primarily associated with recurrent infections, particularly to encapsulated bacteria, and poor antibody responses to polysaccharide vaccines [141]. Mutations in TACI are the most common molecular defects observed in CVID, with approximately 10% of CVID patients carrying a mutation in *TNFRSF13B*, predominantly monoallelic missense variants [142, 143] (Figure 3). The two most common TACI variants identified in CVID are the amino acid substitutions C104R, located in the CRD2 BAFF‐ or APRIL‐binding domain, and A181E, located in the transmembrane domain [144]. C104R inhibits BAFF and APRIL binding and therefore ligand‐induced TACI signaling [145]. While C104R mutant protein is expressed at the cell surface, albeit possibly at decreased levels, and can associate with WT TACI, it does not have a dominant negative effect—rather, heterozygous C104R expression results in TACI haploinsufficiency [141, 144, 145, 146]. The A181E mutation, in contrast, does not inhibit ligand binding but impairs ligand‐induced signaling [145]. A study of knock‐in mice expressing the equivalent murine mutation (A144E) demonstrated that this variant TACI protein is unstable and that A144E heterozygosity in effect reduces TACI surface expression by 50% [117]. Thus, most CVID patients carrying a monoallelic mutation in *TNFRSF13B* are TACI haploinsufficient. Interestingly, 1%–2% of the healthy population also carry monoallelic *TNFRSF13B* variants; thus, TACI mutations are not considered causative but rather a contributing factor that is significantly associated with the development of CVID [144]. A study of a small cohort of healthy heterozygous A181E carriers found that these individuals possessed lower levels of anti‐phosphocholine IgM and IgG antibodies, which provide protection against pneumococcal infection, and lower levels of IgM specific for *E. coli* , indicating that TACI haploinsufficiency may impair TI responses in clinically healthy humans as well as “natural” antibody production [117] (Figure 3).

In light of the newly‐uncovered role for TACI in MZ B cell development, we can now consider that CVID patients with missense mutations in TACI may possess a defect in MZ B cell development that contributes to antibody deficiency. Indeed, the serological profile and defect in antibody responses to polysaccharide antigens is similar between TACI‐mutant CVID patients and TACI‐deficient mice [116, 141]. Similar to mouse MZ B cells, several lines of evidence indicate that human MZ B cells (IgD+ IgM+ CD27+; “unswitched memory”) are also important for antibody responses to such bacterial antigens. Children under 2 years, the elderly and asplenic individuals all exhibit decreased antibody responses against TI infections (e.g., encapsulated bacteria) and polysaccharide vaccinations as well as a decreased frequency of MZ B cells [110, 119]. In addition, while immature MZ B cells can be detected from the foetal stage, the onset of effective TI humoral immunity at the age of ~2 years correlates with the appearance of MZ B cells in the marginal zone of the spleen [113, 147]. Moreover, a reduction in MZ B cells is associated with recurrent respiratory tract infections and reduced anti‐pneumococcal polysaccharide antibodies in CVID patients [148]. Aside from these correlative associations, clonal repertoire analyses have demonstrated that the MZ B cell population is a dominant source of clones that generate anti‐capsular polysaccharide‐secreting PBs and PCs in response to Pneumovax vaccination [110, 118]. Thus, a defective MZ B cell compartment would be expected to result in an impairment of such responses. In support of the hypothesis that development of MZ B cells is impaired in TACI‐mutant CVID patients, reduced proportions of circulating MZ B cells have been observed in a subset of these patients [116, 142]. Nevertheless, detailed phenotypic and functional analyses of MZ B cells in CVID patients are required. As we have seen in TACI‐deficient mice, the numbers of MZ B cells are not necessarily reduced even when their development is impaired, particularly in the presence of elevated BAFF, a phenotype that is also commonly observed in CVID patients [65, 149].

### 11.2 Autoimmunity in TACI‐Associated CVID

Paradoxically, autoimmune manifestations are a frequent clinical symptom in CVID, with a prevalence of ~24%–55% in some CVID populations [143, 144]. Most interestingly, two large cohort studies revealed that CVID patients with monoallelic TACI mutations are more likely to exhibit autoimmune manifestations, particularly autoimmune cytopenias (autoimmune haemolytic anemia, autoimmune thrombocytopenia or Evans syndrome), as well as lymphoproliferation and splenomegaly, compared to CVID patients without TACI mutations [143, 144] (Figure 3). These latter clinical presentations are reminiscent of the B cell hyperplasia and splenomegaly seen in germline TACI‐deficient mice, which we now know are not cell‐intrinsic effects of TACI loss, but instead most likely due to increased BAFF levels resulting from the loss of sTACI decoy function [33]. This implies that CVID‐associated TACI missense mutations may similarly result in impaired sTACI decoy function leading to increased serum BAFF levels, thereby promoting B cell survival and a breach of peripheral tolerance [109, 129]. For example, since sTACI protein containing the C104R mutation is unable to bind and sequester BAFF, a heterozygous C104R TACI mutation could result in an increase in serum BAFF levels due to haploinsufficiency in sTACI decoy function. Similarly, heterozygous expression of A181E TACI, which is not expressed at the B cell surface in vivo, may also cause an increase in serum BAFF levels due to a reduction in sTACI decoy. Arguing against this hypothesis is an analysis of serum BAFF concentrations in CVID patients, which found that these individuals have higher BAFF levels on average compared to healthy donors irrespective of whether they carry TACI mutations [149, 150]. Furthermore, BAFF levels were not significantly different between CVID patients with monoallelic versus biallelic TACI mutations. This lack of a significant difference may be due to the large variation in BAFF concentration observed between patients and the small number of TACI‐mutant patients analyzed thus far. Since BAFF levels are negatively correlated with the number of peripheral B cells, it may be more informative to interpret BAFF concentrations relative to B cell concentrations [65]. This correlation between the two may be altered in people carrying monoallelic and biallelic TACI mutations, since the number of BAFF‐binding TACI molecules is at least halved.

The dual impact of CVID‐associated TACI missense variants, which likely result in both impaired TACI signaling and reduced sTACI decoy function, may underly the increased susceptibility to splenomegaly, lymphoproliferation and autoimmunity seen in CVID patients with monoallelic TACI mutations compared to those without [143, 144]. Intriguingly, the prevalence of autoimmune manifestations is lower in CVID patients with *biallelic* TACI mutations than in those with monoallelic mutations [116, 144]. Biallelic TACI‐mutant CVID patients also exhibit an absence of circulating ANA, compared to elevated levels in monoallelic TACI‐mutant patients [150]. Together, these observations are in line with the protective effect of TACI deficiency in BAFF‐driven autoimmunity in mice and raise the possibility that complete loss of TACI function is also protective for autoimmune manifestations in human CVID (Figure 3).

### 11.3 BAFF and TACI Balance Immunity and Autoimmunity

As well as being present in ~10% of CVID patients, monoallelic TACI missense mutations are seen in a surprisingly high proportion (1%–2%) of the healthy population [144]. Thus, these mutations are not fully penetrant and additional genetic or environmental factors are likely required for clinical manifestations [144, 151]. This raises the questions of why monoallelic TACI mutations are tolerated in healthy humans and why they are highly prevalent. We postulate that loss of ligand binding ability in 50% of TACI molecules, or a 50% reduction in TACI protein levels, leads to a concomitant increase in serum BAFF levels, which may compensate for loss of TACI by causing a BAFFR‐driven increase in B cell numbers and by promoting the survival of self‐reactive immature B cell clones, thereby expanding the BCR repertoire, which together may improve antibody responses.

A potential clue to the relatively high proportion of TACI mutations in the healthy population comes from a genome‐wide association study of Sardinian individuals, which found that a functional variant in *TNFSF13B* (encoding BAFF) that causes increased BAFF protein expression was likely positively selected in the Sardinian population, possibly as a result of the selective pressure of malaria infection [128]. 45.8% of Sardinians, 13.5% of individuals from other parts of Italy and 3.6% of people in the UK and Sweden carry at least one copy of this BAFF variant allele—the latter similar to the frequency of TACI variants observed in a healthy, predominantly northern European population [128, 144]. Expression of the variant was associated with increased circulating B cells and increased IgG, IgA, and IgM levels [128]. However, the *TNFSF13B* variant was also associated with an increased risk of developing SLE and MS. Thus, increased BAFF may confer a protective advantage for humoral immunity with an associated increased risk of autoimmunity.

TACI variants may confer similar effects. Compared to healthy individuals with no TACI mutations, both CVID patients and healthy donors that carry monoallelic TACI mutations have an increased frequency of self‐reactive B cell clones and higher levels of circulating ANA [150]. These observations indicate that TACI haploinsufficiency is associated with a breach in peripheral B cell tolerance, which could be due to increased BAFF levels. It is intriguing that biallelic TACI‐mutant CVID patients also have an increased frequency of self‐reactive B cell clones and yet have reduced ANA and autoimmune manifestations [144, 150]. As discussed earlier, this indicates that complete loss of TACI function is protective for autoimmunity, perhaps by preventing autoreactive clones from entering the MZ compartment (Figure 3).

Evidence for BAFF‐related compensatory mechanisms beneficial for humoral immunity in the case of TACI haploinsufficiency comes from a study of a small cohort of healthy A181E carriers, which found that although these individuals possessed lower levels of anti‐phosphocholine IgM and IgG antibodies, their phosphocholine‐reactive IgG was of higher affinity compared to that of non‐carriers [117]. It is tempting to speculate that increased BAFF levels in A181E carriers could promote the survival of high‐affinity B cells after engagement with phosphocholine and/or expand the mature repertoire to include higher‐affinity phosphocholine‐reactive B cells due to an impairment in peripheral tolerance. This mechanism for increasing antibody affinity may compensate for reduced antibody responses to TI antigens caused by TACI haploinsufficiency in healthy individuals.

## 12 Therapeutic Targeting of BAFF and APRIL Receptors and Ligands

In view of the essential roles for BAFF, APRIL and their receptors in B cell development, survival and function, a large number of therapeutic agents targeting these proteins have been developed for autoimmune diseases and malignancies where B cells and antibodies are causally linked to pathology.

### 12.1 Blocking Antibodies and Fc‐Fusion Proteins

#### 12.1.1 BAFF

Belimumab is a fully humanized monoclonal antibody that binds to BAFF at the receptor binding pocket as well as the flap region, thereby preventing both its interaction with BAFF receptors as well as the formation of BAFF trimer‐trimers that are required for BAFFR survival signaling [7, 152]. Belimumab therefore neutralizes BAFF and can be used to antagonize B cell survival signaling via BAFFR, and TACI signaling induced by BAFF. Belimumab is FDA‐approved for the treatment of SLE and lupus nephritis and has demonstrated efficacy in large clinical trials, though a substantial proportion of patients do not respond [153, 154]. It is not clear if this is due to incomplete B cell depletion, poor tissue penetrance, failure to deplete PCs, which do not depend on BAFF alone for their survival, or patient heterogeneity, or a combination of these factors.

Due to the inverse correlation between B cell numbers and BAFF levels, B cell depletion therapy (BCDT) is followed by an increase in serum BAFF levels [65, 155]. Thus, the combination of BCDT with anti‐BAFF agents such as belimumab is an attractive strategy to prevent the survival of B cell escapees and newly‐generated autoreactive transitional B cell clones. In support of this, treatment with belimumab following rituximab (anti‐CD20) BCDT in SLE led to a reduced incidence of severe flares and levels of anti‐dsDNA IgG, particularly in a subgroup of patients with elevated baseline anti‐dsDNA IgA2 levels [140, 156].

#### 12.1.2 APRIL

*TNFSF13* (encoding APRIL) was identified as a susceptibility locus for IgA nephropathy, in which serum APRIL levels are elevated and correlate with disease‐causing galactose‐deficient (Gd)‐IgA1 levels [157, 158]. Sibeprenlimab, a humanized anti‐APRIL antibody, prevents APRIL binding to its receptors (TACI and BCMA) without affecting BAFF or BAFFR signaling, and thus can block APRIL without causing B cell depletion [159]. Sibeprenlimab reduces IgM and IgA levels (including Gd‐IgA1) more than IgG levels and is efficient at reducing nephropathy symptoms, for example, proteinuria, while preserving SARS‐CoV‐2 vaccine responses [159, 160].

#### 12.1.3 BAFF + APRIL

Given that PBs and PCs rely on either BAFF‐TACI, APRIL‐TACI or APRIL‐BCMA for their maintenance, neutralization of both BAFF and APRIL will be required to inhibit PC survival signaling via TACI and BCMA [122]. Atacicept, telitacicept and povetacicept are recombinant Fc fusion proteins consisting of the wild type or engineered human extracellular TACI domain (CRD1 + CRD2, or CRD2 only in povetacicept), and therefore target both BAFF and APRIL. Atacicept and telitacicept demonstrate efficacy in clinical trials for IgA‐nephropathy and SLE [161, 162, 163, 164, 165]. Interestingly, antibodies to tetanus and diphtheria toxoids largely remain following atacicept treatment, suggesting that vaccine‐induced PCs secreting these antibodies rely on BAFF‐ and APRIL‐independent PC survival mechanisms, and/or there is incomplete BAFF and APRIL neutralization in PC survival niches with atacicept.

#### 12.1.4 BAFFR

Ianalumab is a monoclonal human antibody targeting BAFFR that both blocks BAFFR binding to BAFF and mediates B cell depletion via antibody‐dependent cellular cytotoxicity (ADCC). Ianalumab has demonstrated efficacy in Phase 2 trials for SjD and SLE [166, 167].

### 12.2 Bispecific T Cell Engagers (BiTEs)

The CD3‐BCMA bispecific antibodies aim to crosslink cytotoxic T cells and BCMA‐expressing multiple myeloma (MM) cells to achieve tumor cell lysis, without the need for lymphodepletion or generation of autologous CAR T cells. Teclistamab has demonstrated encouraging results in early case series for the treatment of refractory autoimmune diseases but has not yet entered clinical trials for these indications [168, 169, 170].

### 12.3 Chimeric Antigen Receptor T Cell (CAR T) Therapy

Two CAR T therapies are currently approved for the treatment of MM that target BCMA: ide‐cel and cilta‐cel [171, 172]. Anti‐BCMA CAR T has also demonstrated efficacy in patients with relapsed/refractory neuromyelitis optica spectrum disorder, with a majority of patients becoming seronegative for anti‐AQP4 IgG. Furthermore, dual‐specificity CAR T targeting both CD19 and BCMA, or co‐infusion of anti‐CD19 and anti‐BCMA CAR T, has led to disease remission and autoantibody negativity in SLE and refractory myasthenia gravis in Phase I clinical trials [173, 174, 175, 176]. Given that long‐lived PCs lose CD19 expression, therapies targeting BCMA may be beneficial for autoimmune disease driven by long‐lived autoreactive PCs [126].

## 13 Concluding Remarks

BAFF and APRIL and their three related receptors form a system with both unique and redundant functions essential for B cell and PC biology. While only BAFF and BAFFR are able to support FO and MZ B cell survival, PC maintenance is dependent on either BAFF or APRIL and either TACI or BCMA. In addition to their roles in cell survival, it is now clear that these ligands and receptors also support B cell development, as exemplified by the role of TACI in MZ B cell development.

These findings leave a number of open questions. Why does the survival of FO and MZ B cells switch from depending on BAFFR to requiring TACI or BCMA when they differentiate into PC? BAFFR signaling is less potent without surface BCR [55], so PCs, which no longer express BCRs, may be unable to survive using BAFFR and thus lose BAFFR expression replacing it with TACI and BCMA. Is this also to allow independent control of numbers of B cells and PCs? If both depended on the same survival factor and signaling pathway, that is, BAFF and BAFFR, they might compete with each other for survival signals. Thus, the switch from dependence on BAFFR to dependence on TACI and BCMA, which can bind APRIL, may lead to independently regulated pools of cells. On a similar issue, we note that MBCs require BAFF and BAFFR for their survival so would be expected to compete with the much larger number of naive FO and MZ B cells, yet MBCs are able to persist and some have much longer half‐lives compared to naive B cells. How is this achieved? Are MBCs more sensitive to BAFF‐BAFFR signals, surviving on lower levels of BAFF? Do they use additional survival signals?

Why are both TACI and BCMA able to support PC survival? Are there subtypes of PC that depend more on one of these receptors? Or are they used differentially in distinct anatomical locations within different survival niches? Proteolytic cleavage of both TACI and BCMA generates sTACI and sBCMA proteins that likely act as decoy receptors for BAFF and/or APRIL. The observation that loss of TACI results in increased levels of BAFF, presumably due to loss of sTACI decoy function, leads to the question of why such a function has evolved. It may act as a feedback circuit to regulate the number of B cells—a circuit that is disrupted by common TACI missense variants. However, to date no function has been identified for sBCMA. Finally, while we now know that TACI supports MZ B cell development, it is not known if other B cell subsets require signals from any of the three receptors for their maturation.

Future studies addressing these questions will need to take into account these feedback circuits when designing loss‐of‐function experiments to avoid confounding effects. Additionally, it will be important to carry out mutational analyses using inducible systems in order to clearly distinguish survival and differentiation functions. Finally, the mechanisms by which the three receptors signal are incompletely understood and require further study. The outcome will be a deeper understanding of the functions of this critical receptor system, which remains a major therapeutic focus.

## Funding

This work was supported by the Francis Crick Institute (CC2080) that receives its core funding from the Medical Research Council (CC2080), Wellcome Trust (CC2080), and Cancer Research UK (CC2080).

## Conflicts of Interest

The authors declare no conflicts of interest.

## Data Availability

Data is available upon request.
